# WISP1 and TLR4 on Macrophages Contribute to Ventilator-Induced Lung Injury

**DOI:** 10.1007/s10753-019-01103-0

**Published:** 2020-03-04

**Authors:** Zhuang Yu, Tingting Wang, Liming Zhang, Xiaohu Yang, Quan Li, Xibing Ding

**Affiliations:** 1 Department of Anesthesiology, Shanghai General Hospital, Jiaotong University School of Medicine, Shanghai, China; 2 Department of Anesthesiology, Huashan Hospital, Fudan University School of Medicine, Shanghai, China; 3 Department of Anesthesiology, University of Pittsburgh School of Medicine, Pittsburgh, PA USA; 4 Department of Anesthesiology, Shanghai East Hospital, Shanghai, China; 5 Department of Anesthesiology, Cancer Hospital Chinese Academy of Medical Sciences, Shenzhen, China; 6 Department of Anesthesiology, Shanghai Renji Hospital, Jiaotong University School of Medicine, Shanghai, China

**Keywords:** WISP1, TLR4, mechanical ventilation, lung injury

## Abstract

Injurious mechanical ventilation has been shown to directly affect pulmonary and systemic immune responses. How these responses propagate or attenuate remains unknown. The goal of this study was to further determine whether toll-like receptor (TLR) 4 and WNT1-inducible signaling pathway protein 1 (WISP1) could contribute to injurious mechanical ventilation, especially focusing on the role of macrophages during experimental ventilator-induced lung injury. A prospective, randomized, and controlled animal study was designed, and male, wild-type (WT) C57BL/6 mice, TLR4 knockout (TLR4^−/−^), and lyzTLR4 knockout (lyzTLR4^−/−^) mice aging 8~12 weeks were used. Animals were anesthetized and randomized to spontaneous breathing (SB) group or to high tidal volume (VT, 20 ml/kg) mechanical ventilation (HTV) group. Histological evaluation, alveolar–capillary permeability of Evan’s blue albumin (EBA), WISP1 protein levels, macrophage inflammatory protein-2 (MIP-2), and interleukin-6 (IL-6) in plasma and bronchoalveolar lavage fluid (BALF) concentrations were analyzed. HTV group was associated with a significant increase of WISP1 and EBA ratio in C57BL/6 mice, a significant decrease of WISP1 protein levels, and a significant decrease of IL-6, MIP-2 in plasma, and BALF concentrations of pro-inflammatory cytokines in TLR4^−/−^ and lyzTLR4^−/−^ knockout mice. In TLR4^−/−^ mice and lyzTLR4^−/−^ mice, there were also significant differences between SB group and HTV group in terms of H&E score and EBA ratio and level of pro-inflammation cytokines. The entire TLR4-targeted mice could further improve various inflammatory changes and damages when compared with lyzTLR4-targeted mice. What is more, TLR4^−/−^ mice and lyzTLR4^−/−^ mice reacted differently to rWISP1 and/or BMMC treated. TLR4^−/−^ mice had no response to rWISP1, while lyzTLR4^−/−^ mice still showed drastic response to both treatments. TLR4 and WISP1, especially the former one, on macrophages could contribute to releasing of pro-inflammatory cytokines during ventilator-induced lung injury. Injurious mechanical ventilation may result in an immune response which is similar to that of infection.

## INTRODUCTION

There are a lot of studies suggesting that lung overdistension during mechanical ventilation (MV) causes or exacerbates lung injury [1, 2]. Although mechanical ventilation is an important part of supportive therapy in the management of acute lung injury, such therapy may produce an iatrogenic complication referred to as ventilator-induced lung injury (VILI); this condition may be difficult to diagnose clinically because its symptoms may be similar to the damage caused by the primary lung disease [3–5].

Several studies have demonstrated that certain MV strategies may lead to synthesis and release of pro-inflammatory cytokines, such as IL-6 and MIP-2, from the lung soon after initiation of MV, which contributes to the development of a systemic inflammatory response that produces or aggravates lung damage and may lead to multiple organ failure [6, 7].

Most pulmonary cells express a large variety of genes under transcriptional control that are mediated by biomechanical forces [8, 9] and bacterial/viral infections [10]. Essential components of the innate immune system are the toll-like receptors (TLRs) [11, 12], especially TLR4 [6, 13, 14] which has been regarded to match microbial products and join in VILI. Previously, our study has identified that WNT1-inducible signaling pathway protein 1 (WISP1), by genome-wide association study (GWAS), would contribute to VILI [15]. What is more, we also reported that TLR4 and WISP1 combined to aggravate liver warm ischemia-reperfusion injury and non-infected inflammation response [16]. But it remains unknown whether TLR4 and WISP1 could co-contribute to VILI.

Held *et al*. [17] reported that inflammatory responses induced by HTV MV were similar to those evoked by LPS, and another research reported that macrophage deletion could effectively attenuate TLR4-dependent VILI [18]. Therefore, we hypothesized that MV could modulate the WISP1/TLR4 signaling pathway in macrophages in VILI. We evaluated this hypothesis by establishing suitable animal HTV models of VILI, and then test relevant factors to confirm it.

## METHODS AND MATERIALS

### Animals

Experimental animal protocols were performed in accordance with guidelines approved by the Institutional Animal Care and Use Committee at the University of Pittsburgh (Pittsburgh, PA). Wild-type (WT) C57BL/6 mice, TLR4 knockout (TLR4^−/−^) mice, and lyzTLR4 knockout (lymphatic myeloid mononuclear cells, lyzTLR4^−/−^) mice, aging 8~12 weeks, were anesthetized (100 mg/kg ketamine and 10 mg/kg xylazine) and ventilated with HTV (20 ml/kg body weight, 100 breaths/min, 4 h; 0 positive end-expiratory pressure) to establish VILI models.

### Alveolar–Capillary Permeability of Evan’s Blue Albumin (EBA)

Alveolar–capillary permeability was estimated with EBA according to our previous studies [19, 20]. EBA (25 mg/kg body weight) was administrated through the vena jugularis externa 1 h before sacrificing all models. Blood samples were obtained from the right heart, and the pulmonary vasculature was subsequently infused with 1 ml PBS. The right lung was excised, cleaned, weighed, and preserved in liquid nitrogen until these samples were used for EBA analysis.

### Histological Examination

The appropriate lobe lung of each model was taken to make histological hematoxylin eosin (H&E) staining. Lung sections were scored for lung injury, including the following: (1) alveolar and capillary edema, (2) intravascular and peribronchial influx of inflammatory cells, (3) thickness of the alveolar wall, and (4) hemorrhage. All items were semi-quantitatively scored as none, minimal, light, moderate, or severe (score 0, 1, 2, 3, or 4, respectively) by a pathologist blinded to the experimental group.

### Enzyme-Linked Immunosorbent Assay (ELISA)

All samples of plasma and BALF were prepared in order to quantify the levels of secreted cytokines. Cytokines of IL-6 (R&D, M6000B, USA) and MIP2 (R&D, MM200, USA) concentrations were measured using a specific ELISA assays.

### SDS-PAGE and Western Blot Analysis

Proteins were extracted from the frozen lung tissues by grinding with protease inhibitors. The proteins were incubated on ice for 30 min. The protein samples were separated by 10% sodium dodecyl sulfate polyacrylamide gel electrophoresis (SDS-PAGE) and transferred onto a polyvinylidene fluoride (PVDF) membrane. The membranes were incubated with 5% non-fat milk for 1 h, and then incubated with primary antibodies. WISP1 of primary rabbit monoclonal antibody (ab178547, Abcam, USA) was used for Western blot analysis. Membranes were detected using the Odyssey Two-Color Infrared Laser Imaging System (LI-COR Biosciences).

### Bone Marrow–Derived Macrophage Cells (BMMCs)

For BMMC purification, femurs and tibias were taken from sacrificed WT mice and flushed with a 1 ml syringe filled with Hank’s balanced salt solution (HBSS, Life Technologies, 14170112) containing 0.1% bovine serum albumin and 20 mM HEPES (pH 7.4). Following RBC lysis, the BM cell suspensions were filtered by a 40-μm cell strainer (Falcon). Then, it was stimulated with complete medium containing GM-CSF for 7 days.

### Statistical Analysis

Data are expressed as means ± standard error of the mean (SEM). Statistically significant differences (^*^P < 0.05, ^**^P < 0.01, ^***^P < 0.001) were determined by two-way or one-way ANOVA, followed by Bonferroni’s multiple comparisons or Tukey’s post-test, respectively, using GraphPad Prism ver. 5.0 (GraphPad software, San Diego, CA). The significant level was set at 0.05.

## RESULTS

### TLR4 Significantly Promoted HTV-Induced Lung Injury

**Fig. 1:**
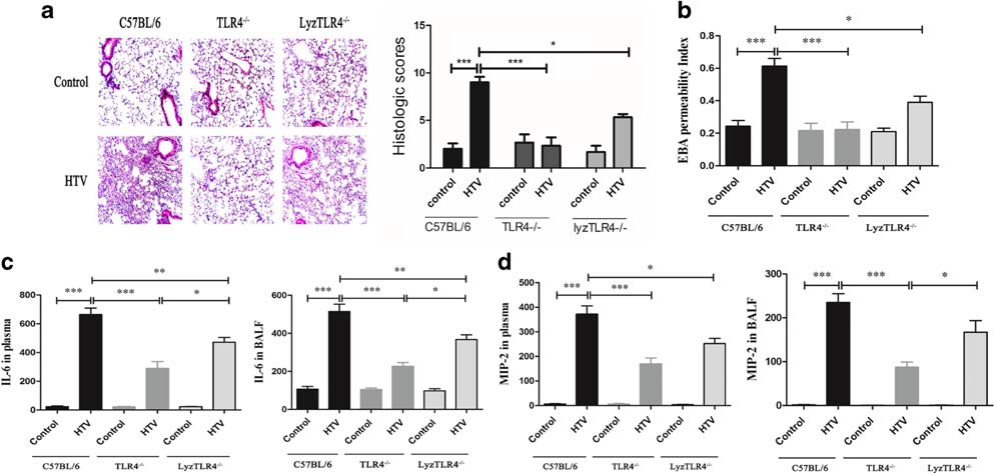
TLR4 significantly promotes HTV-induced lung injury. WT mice and different levels of TLR4-targeted mice were treated with or without HTV. **a** Lung H&E staining and the histological alterations of lung parenchyma were shown about graded on a scale from 0 to 4. **b** EBA depicts the permeability of alveolar capillary in each group. ELISA was performed with the standard protocol, and cytokine profiles of IL-6 and MIP-2 (**c**, **d**) were tested at all control and HTV groups (^*^P < 0.05; ^**^P < 0.01; ^***^P < 0.001).

Because structural damage in the alveolar–capillary membrane barrier with subsequently increased pulmonary vascular permeability is a prominent feature of acute lung injury, we used the ratio of EBA levels after HTV and EBA levels after spontaneous breathing of each group as a quantitative parameter to discriminate lung injury after mechanical ventilation in each mouse strain. As shown in Fig. 1b, the EBA permeability ratio of WT mice after HTV increased roughly threefold (*P* < 0.001). What is more, in terms of EBA permeability ratio after HTV, the TLR4^−/−^ mice had the lowest level (*P* < 0.001), followed by lyzTLR4^−/−^ mice (*P* < 0.05) compared with WT mice. Accordingly, lung H&E staining (Fig. 1a) showed a similar result. We observed more structural damage, inflammatory cell infiltration, and interstitial thickening after HTV in WT mice, while the levels of those factors were significantly lower in TLR4^−/−^ mice (*P* < 0.01) and lyzTLR4^−/−^ mice (*P* < 0.05) when compared with WT mice.

High VT MV also upregulated IL-6 and MIP-2 levels in the plasma and BALF of WT mice (*P* < 0.001). Similarly, although MV with a HTV of 20 ml/kg significantly changed IL-6 and MIP-2 levels, there was also an improvement in both TLR4^−/−^ mice and lyzTLR4^−/−^ mice. And we also found that TLR4^−/−^ mice and lyzTLR4^−/−^ mice (*P* < 0.01) showed significant alleviation (*P* < 0.001) when compared with WT mice (Fig. 1c, d).

### TLR4 Could Mediate WISP1 Expression Level in VILI

**Fig. 2:**
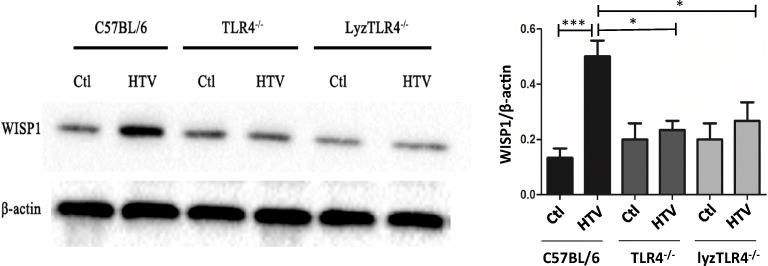
TLR4 would mediate WISP1 expression level in VILI. Western blotting shows the effects of mechanical ventilation on WISP1 protein levels in several groups of HTV animals *vs* control groups: spontaneous breathing and ventilated with high tidal volume (among C57, TLR4^−/−^, lyzTLR4^−/−^ sub-groups) for 4 h (^*^P < 0.05, ^**^P < 0.01, ^***^P < 0.001).

As shown in Fig. 2, the Western blot apparently exhibited that WISP1 was upregulated after 4-h HTV in WT mice (*P* < 0.001). In contrast, WISP1 expression level in TLR4^−/−^ mice and lyzTLR4^−/−^ mice after HTV remained the same as that in control group. However, there was no difference between TLR4^−/−^ mice and lyzTLR4^−/−^ mice in terms of WISP1 level after they were induced by HTV.

### rWISP1 Could Accelerate Inflammatory Injury in WT and lyzTLR4-Targeted But Not Entire TLR4-Targeted

**Fig. 3:**
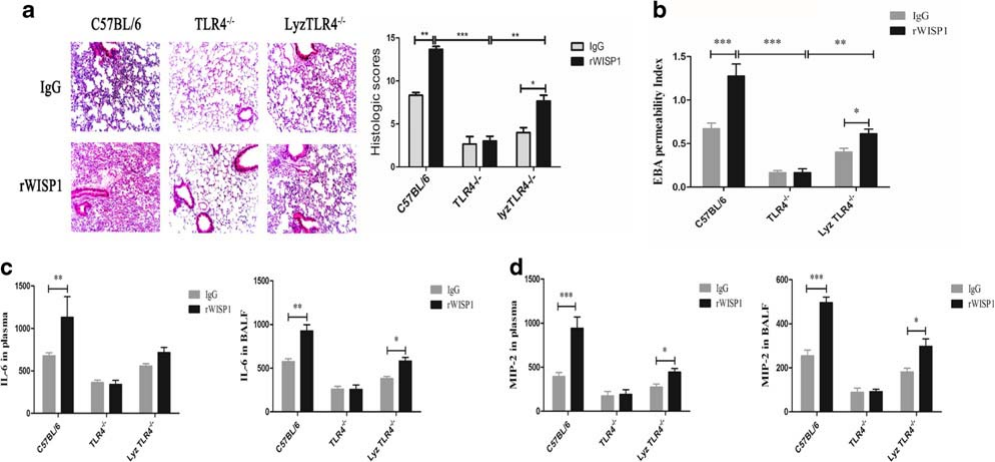
rWISP1 could accelerate inflammatory injury in WT and lyzTLR4-targeted but not entire TLR4-targeted*.* Isotype control IgG via intratracheal (i.t.) injection together with HTV or rWISP1 protein (20 μg per mouse) i.t. **a** Lung H&E staining shows structure changes among different groups. **b** EBA shows the alveolar–capillary permeability after HTV with/without rWISP1. Cytokine profiles of IL-6 and MIP-2 (**c**, **d**) were tested by ELISA after HTV with/without rWISP1 (^*^P < 0.05; ^**^P < 0.01; ^***^P < 0.001).

rWISP1 protein (20 μg/mouse) via intratracheal (i.t.) injection together with HTV, or isotype control IgG i.t., has been applied. As shown in Fig. 3b, rWISP1 could increase the EBA ratio after HTV in WT mice (*P* < 0.001), and both TLR4-targeted groups showed significant improvement. In addition, there was also a significant difference between TLR4^−/−^ mice and lyzTLR4^−/−^ mice (*P* < 0.05). WISP1 could also reverse the decrease of lyzTLR4^−/−^ mice EBA ratio (*P* < 0.05) after HTV to some degree, but it could not change that of TLR4^−/−^ mice. Meanwhile, lung H&E staining (Fig. 3a) also demonstrated a similar outcome; lungs from groups ventilated with HTV showed acute inflammatory infiltration and perivascular edema in WT mice with rWISP1 administrated than WT with IgG (*P* < 0.001) and TLR4-targeted with rWISP1 (*P* < 0.001 when compared with TLR4^−/−^ mice, *P* < 0.01 when compared with lyzTLR4^−/−^ mice). However, no major histological difference was observed in TLR4^−/−^ mice with rWISP1 or IgG, while lyzTLR4^−/−^ mice still had a significance between rWISP1 and IgG sub-groups (*P* < 0.05).

In addition, rWISP1 administrated could result in a marked change on IL-6 (*P* < 0.01) and MIP-2 (*P* < 0.001) levels after HTV. Meanwhile, cytokine profiles were similar in animals ventilated with HTV and rWISP1 treated in TLR4^−/−^ mice. But IL-6 and MIP-2 levels markedly increased in both BALF and plasma in all lyzTLR4^−/−^ sub-group samples. However, the increase was relatively lower in lyzTLR4^−/−^ mice when compared with WT. All this demonstrated that TLR4 was not only on macrophages but also on other cells, and it could combine WISP1 to enhance inflammatory reactions (Fig. 3c, d).

### rWISP1 and TLR4 on BMMC-Driven Inflammation and Lung Injury After HTV

As shown in Fig. 3, there was no significant change in TLR4^−/−^ mice. These images indicated that an extreme situation like entirely TLR4-targeted could completely diminish the effects of WISP1 on pro-inflammation. Based on this phenomenon, we tried to further investigate if macrophages, as a bridge, could combine TLR4 and WISP1 to deteriorate all these changes.

**Fig. 4:**
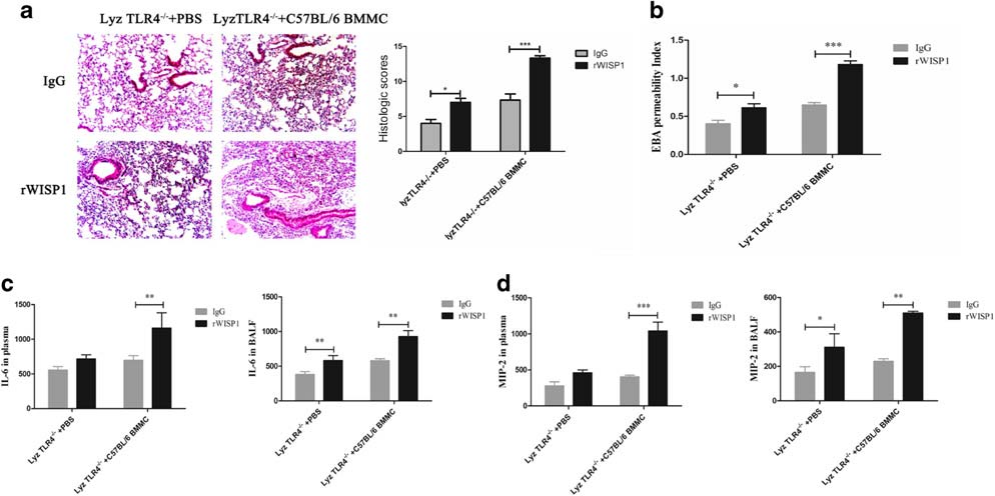
rWISP1 and TLR4 on BMMC-driven inflammation and lung injury after HTV. LyzTLR4^−/−^ mice were chosen and injected with BMMC (10^6^/ml) and/or rWISP1 from respiratory tract. **a** Lung H&E staining shows structure changes among different groups. **b** EBA depicts the permeability of alveolar capillary in each group with different methods tackled. LyzTLR4^−/−^ mice were chosen and injected with BMMC (10^6^/ml) and/or rWISP1 from respiratory tract. Finally, IL-6 and MIP-2 concentrations were tested in plasma and BALF (**c**, **d**) (^*^P < 0.05; ^**^P < 0.01; ^***^P < 0.001).

LyzTLR4^−/−^ mice were chosen and injected with BMMC (10^6^/ml) from respiratory tract. The results showed that lyzTLR4^−/−^ mice which were treated with both rWISP1 and BMMC had the highest level of alveolar–capillary permeability (Fig. 4b) and the worst pathophysiologic evaluations (Fig. 4a). These indicated that TLR4 on macrophages played an interactive and amplified role in collaborating with WISP1 effects.

We further tested IL-6 and MIP-2 concentration in plasma and BALF, and the results are shown in Fig. 4c, d. As expected, rWISP1 given alone to LyzTLR4^−/−^ mice with HTV could upregulate MIP-2 and IL-6 levels in BALF (*P* < 0.05) but caused no change in plasma. In addition, BMMC given alone caused no changes in both BALF and plasma. Above of all, these phenomena may indicate that rWISP1 could exaggerate the local inflammation after HTV when TLR4 existed no matter on macrophages or not; in addition, the results could also turn out that TLR4 on macrophages provided a potential main function to inflammation responses.

## DISCUSSION

It was found in this study that HTV MV, in the absence of infection, with different levels of TLR4-targeted mice and treatments could result in different impacts on pro-inflammatory cytokine levels, such as IL-6 and MIP-2, in the BALF and in the systemic circulation. These findings suggested that inappropriate MV may represent a stimulus for the immune system similar to that caused by severe bacterial infections. Meanwhile, our study also revealed that TLR4 on macrophages and WISP1 might have a cooperative relationship during the response procedure to VILI.

It has been reported previously that synergy between viral and bacterial TLR signaling could lead to amplification of the inflammatory response [11, 12, 21]. Mechanical ventilation, which is a component of supportive therapy in the management of acute lung injury, can also produce an iatrogenic complication referred to as ventilator-induced lung injury (VILI). Possible mechanism has been elucidated by several researches, which reported innate immunity TLR signaling combined with CD14 [15, 22] or MyD88 [20, 23] or IRAK3 [14, 24] to participate in VILI. Despite the finding that low volume tidal (LV_T_) strategy may be less injurious or even therapeutic for patients who need MV support [24], another research suggested that LV_T_ could also induce lung inflammation and increase the risk of VILI [20, 23, 25, 26]. These injurious results were also resulted from TLR signaling regulation. Meanwhile, the main emphasis on TLR4 in VILI was its downstream NF-kB pathway in the researches of Dai *et al*. and Villar *et al*. [23, 24]. Their studies suggested that TLRs were the triggers for inflammatory response caused by MV in healthy lungs [14, 23], which was in line with our results (Fig. 3). Both IL-6 and MIP-2 had no significance in all TLR4^−/−^ samples treated with rWISP1 after HTV. This indicated that WISP1 could promote cytokine secretion procedure only when TLR4 existed. The study of Kuhn et al. [6] suggested that TLR4-mediated innate immunity increased cell damage induced by stretch, but it did not modify TLR4-mediated innate immunity. This finding gave alternative hypothesis that VILI initiated TLR4 mediating immune response, and then, it further amplified inflammatory cytokine-secreted cascades, contributing to complications which had symptoms overlapped with primary diseases.

MV can mediate the innate immune response to HTV ventilation by inducing the production of NF-kB and pro-inflammatory cytokine, which is consistent with prior reports. Gene expression profiles obtained from microarrays across different experimental models of VILI also suggested that the response triggered by alveolar overdistension might imitate an innate immune inflammatory response against pathogens [27–29]. As our previous work reported, WISP1 [15], identified by a genome-wide approach, acted as an affiliated adaptor molecule that contributed to VILI in mice through controlling and/or amplifying TLR4-mediated cellular functions, which was confirmed in our present study. The entire TLR4^−/−^ mice with rWISP1 administration could not increase IL-6 and MIP-2 concentration, suggesting that WISP1 could not affect local or systematic inflammation reaction without TLR4. In addition, we also found that pure over-distension on healthy mice lung could only raise WISP1 level, while it could not upregulate cytokine levels (the results were not shown in this paper). Reports about WISP1 mainly concentrated on WISP1/β-catenin signaling pathway[30], like our previous study about WISP1 interacting with β6 [19, 31] or with β3 [32, 33], which was related to alveolar–capillary permeability in VILI or ALI/ARDS.

In this study, we used entire body TLR4^−/−^ mice and lyzTLR4^−/−^ mice to establish that exactly distinguished TLR4 on macrophages to establish two completely different HTV VILI models. Although BMMC lyzTLR4^−/−^ mice model was used in this study, we still cannot conclude that only TLR4 on macrophages can interact with WISP1. β-Integrin family members or other factors might also play a similar role in these inflammation reactions. We will further investigate them in our latter work. Of note, we found that there was significant difference in terms of lung injury degree between TLR4^−/−^ and lyzTLR4^−/−^ mice after HTV, which indicated that entire body TLR4 knockout could relieve VILI. This finding potentially provides strategy for producing TLR4 blocked drugs for the whole body. In addition, it indicated that TLR4 may also exist on other types of cells in lung tissue, such as alveolar cells, and then facilitate the inflammatory response [6]. In our previous study, we had confirmed that WISP1/TLR4 signaling pathway could aggravate liver warm ischemia-reperfusion injury and non-infected inflammation response [16], and it was also confirmed in this study that TLR4 could mediate expression level of WISP1 (Fig. 2) in VILI. However, how TLR4 and WISP1 interact with each other is still unrevealed. Whether MV induces lung injury via WISP1/TLR4 signaling combined with macrophage still requires further study.

Although HTV is not normally used in clinic, establishing VILI model for mechanism study is still necessary. More investigations are needed to explain how VILI happens, develops, and outcomes. New therapeutic approaches and strides are expected in the future.
